# Rational design of a covalent ACE2 decoy receptor that broadly neutralizes SARS‐CoV‐2 variants

**DOI:** 10.1002/pro.70306

**Published:** 2025-09-13

**Authors:** Nobumasa Hino, Risa Takada, Kyosuke Suzuki, Nagisa Tokunoh, Tatsuya Karaki, Kazumasa Ohtake, Haruna Ogami, Takafumi Nishiura, Kenji Ishimoto, Tomohito Tsukamoto, Yukio Ago, Yoshiaki Okada, Toru Okamoto, Chikako Ono, Yoshiharu Matsuura, Satoshi Obika, Kensaku Sakamoto, Yasuo Yoshioka, Shinsaku Nakagawa

**Affiliations:** ^1^ Laboratory of Biopharmaceutics, Graduate School of Pharmaceutical Sciences The University of Osaka Osaka Japan; ^2^ Laboratory of Bioorganic Chemistry, Graduate School of Pharmaceutical Sciences The University of Osaka Osaka Japan; ^3^ Center for Infectious Disease Education and Research The University of Osaka Osaka Japan; ^4^ Vaccine Creation Group, BIKEN Innovative Vaccine Research Alliance Laboratories, Research Institute for Microbial Diseases The University of Osaka Osaka Japan; ^5^ The Research Foundation for Microbial Diseases of Osaka University Osaka Japan; ^6^ Laboratory for Nonnatural Amino Acid Technology, RIKEN Center for Biosystems Dynamics Research Kanagawa Japan; ^7^ Laboratory for Advanced Biomolecular Engineering, RIKEN Center for Integrative Medical Sciences Kanagawa Japan; ^8^ Department of Electrical Engineering and Bioscience Waseda University Tokyo Japan; ^9^ Global Center for Medical Engineering and Informatics The University of Osaka Osaka Japan; ^10^ Department of Cellular and Molecular Pharmacology, Graduate School of Biomedical and Health Sciences Hiroshima University Hiroshima Japan; ^11^ Laboratory of Clinical Science and Biomedicine, Graduate School of Pharmaceutical Sciences The University of Osaka Osaka Japan; ^12^ Institute for Advanced Co‐Creation Studies, Research Institute for Microbial Diseases Osaka University Osaka Japan; ^13^ Department of Microbiology Juntendo University School of Medicine Tokyo Japan; ^14^ Laboratory of Virus Control, Research Institute for Microbial Diseases The University of Osaka Osaka Japan; ^15^ Center for Advanced Modalities and DDS The University of Osaka Osaka Japan; ^16^ Department of Drug Target Protein Research Shinshu University School of Medicine Nagano Japan; ^17^ Laboratory of Nano‐Design for Innovative Drug Development, Graduate School of Pharmaceutical Sciences The University of Osaka Osaka Japan; ^18^ Vaccine Creation Group, BIKEN Innovative Vaccine Research Alliance Laboratories, Institute for Open and Transdisciplinary Research Initiatives The University of Osaka Osaka Japan

**Keywords:** ACE2‐Fc, covalent proteins, cross‐linking, genetic code expansion, non‐canonical amino acids, SARS‐CoV‐2

## Abstract

The ongoing evolution of severe acute respiratory syndrome coronavirus 2 (SARS‐CoV‐2) and the emergence of variants that evade existing vaccines and antibody therapies necessitate novel, potent, and broad‐spectrum antiviral strategies. Enhancing therapeutic proteins with additional covalent binding capabilities, such as minibinders and nanobodies, reportedly potentiates their antiviral efficacy by irreversibly capturing the viral cell‐entry protein. However, viral mutations that interfere with covalent bonding or reduce viral affinity with therapeutic proteins might compromise the efficacy of this strategy. Therefore, in this study, we aimed to develop a broadly neutralizing covalent angiotensin‐converting enzyme 2‐Fc (ACE2‐Fc) decoy using a rational design strategy that integrates functional genomics with structural information. Using this approach, we targeted a highly conserved and functionally constrained residue on the viral receptor‐binding domain (RBD) and identified tyrosine 473 (Y473) as an optimal target. We engineered ACE2‐Fc constructs by replacing glutamate 23 (E23) and threonine 27 (T27) with the non‐canonical amino acid—fluorosulfate‐L‐tyrosine (FSY), generating E23FSY and T27FSY constructs. These constructs formed a specific and efficient covalent bond with Y473 of the RBD. Notably, this covalent capture was retained against the highly mutated Omicron BA.5 RBD. In pseudovirus neutralization assays, both E23FSY and T27FSY exhibited markedly enhanced potency against both wild‐type‐like (D614G) and Omicron variants compared to their non‐covalent counterparts. These results demonstrate that using an inherently escape‐resistant decoy receptor to covalently target evolutionarily constrained residues on the viral RBD is a highly efficient strategy for creating potent, broad‐spectrum covalent inhibitors against rapidly evolving viruses such as SARS‐CoV‐2.

## 1 INTRODUCTION

Coronavirus disease 2019 (COVID‐19), caused by the severe acute respiratory syndrome coronavirus 2 (SARS‐CoV‐2), has resulted in >7 million deaths worldwide since its emergence in late 2019 (World Health Organization, n.d.; Hu et al., 2021; V'Kovski et al., 2021). Both messenger RNA vaccines and virus‐neutralizing antibody therapeutics have been developed at an unprecedented pace. However, the continuous evolution of the virus and the resulting emergence of variants of concern (VOCs) have enabled immune evasion following both natural infection and vaccination, posing a persistent and ongoing challenge to public health (Cao et al., 2022; Harvey et al., 2021). The primary antigenic site on the surface of the SARS‐CoV‐2 virion is the spike (S) protein trimer, which mediates virus‐host membrane fusion and entry via the primary host cell receptor, angiotensin‐converting enzyme 2 (ACE2) (Hoffmann et al., 2020; Walls et al., 2020). Viral entry is initiated by the specific interaction of the receptor‐binding domain (RBD) of the S1 subunit with ACE2, followed by membrane fusion facilitated by the S2 subunit. Most neutralizing antibodies elicited by natural infection or vaccination block this interaction (Barnes et al., 2020). However, immune selection pressure leads to viral escape mutations, often generating VOCs with enhanced receptor binding or antibody evasion, thereby reducing the efficacy of existing neutralizing antibodies (Garcia‐Beltran et al., 2021; Starr et al., 2021).

An alternative to neutralize SARS‐CoV‐2 involves the use of soluble ACE2 (sACE2) decoys derived from the ectodomain of ACE2 (Monteil et al., 2020). In principle, viral mutants that escape ACE2 decoys would simultaneously lose their ability to bind endogenous, cell‐surface ACE2, making this approach inherently resistant to escape. However, the relatively low affinity of wild‐type (WT) ACE2 for the RBD—dissociation constant (Kd) in the range of 15–50 nM (Hoffmann et al., 2020; Monteil et al., 2020; Walls et al., 2020)—has limited the therapeutic potential of native sACE2. Therefore, the binding affinities of sACE2 and its Fc‐fused form (ACE2‐Fc) have been considerably improved to the sub‐nanomolar range through methods such as deep mutagenesis (Chan et al., 2020; Chan et al., 2021), computational design (Glasgow et al., 2020; Havranek et al., 2021, 2023), and directed evolution (Higuchi et al., 2021; Ikemura et al., 2022; Iwanaga et al., 2022), either individually or in combination. Although these engineered high‐affinity ACE2 decoys represent promising therapeutics against SARS‐CoV‐2 variants, their binding to the S protein remains reversible. This reversibility necessitates high or sustained concentrations for effective neutralization and may not ensure complete viral inactivation.

To address this limitation, strategies employing proximity‐enabled covalent chemistry have emerged, aiming to achieve irreversible target inactivation. One such approach uses genetic code expansion to incorporate non‐canonical amino acids (ncAAs) bearing latent bioreactive groups, such as fluorosulfate‐L‐tyrosine (FSY) and fluorine‐substituted fluorosulfate‐L‐tyrosine (FFY), into therapeutic protein candidates (Han et al., 2022; Li et al., 2020; Wang et al., 2018; Yu et al., 2022). Upon target binding, the fluorosulfate group is positioned close to reactive residues on the target protein, enabling FSY or FFY to form stable covalent bonds with nearby histidine, lysine, or tyrosine residues via sulfur‐fluoride exchange (SuFEx) chemistry. This proximity‐dependent covalent modification strategy has been applied to various anti‐RBD protein scaffolds, including minibinders and nanobodies, and has shown promise in enhancing viral neutralization (Han et al., 2022; Yu et al., 2022).

However, a pronounced challenge of this approach is its reliance on the initial non‐covalent binding of the protein scaffold. This vulnerability has been observed in practice; the cross‐linking efficiency of FSY‐ and FFY‐incorporated minibinders and nanobodies decreases when targeting highly mutated Omicron sublineage RBDs due to the reduced binding affinity of their artificial scaffolds (Han et al., 2022; Yu et al., 2022). Even when using a decoy receptor, which is inherently more resistant to viral escape, targeting a mutation‐prone residue such as lysine 417 (K417) resulted in a complete loss of covalent capture against Beta and Omicron variants harboring the K417N mutation (Yu et al., 2022). This study aimed to overcome these limitations by developing a broadly neutralizing covalent ACE2‐Fc decoy that combines a mutation‐resistant native receptor scaffold with structure‐guided site‐specific incorporation of FSY at positions predicted to target functionally critical and evolutionarily conserved RBD residues.

## 2 RESULTS

### 2.1 Tyrosyl‐tRNA synthetase (TyrRS) variants incorporate FSY more efficiently than pyrrolysyl‐tRNA synthetase (PylRS)‐based systems

**FIGURE 1:**
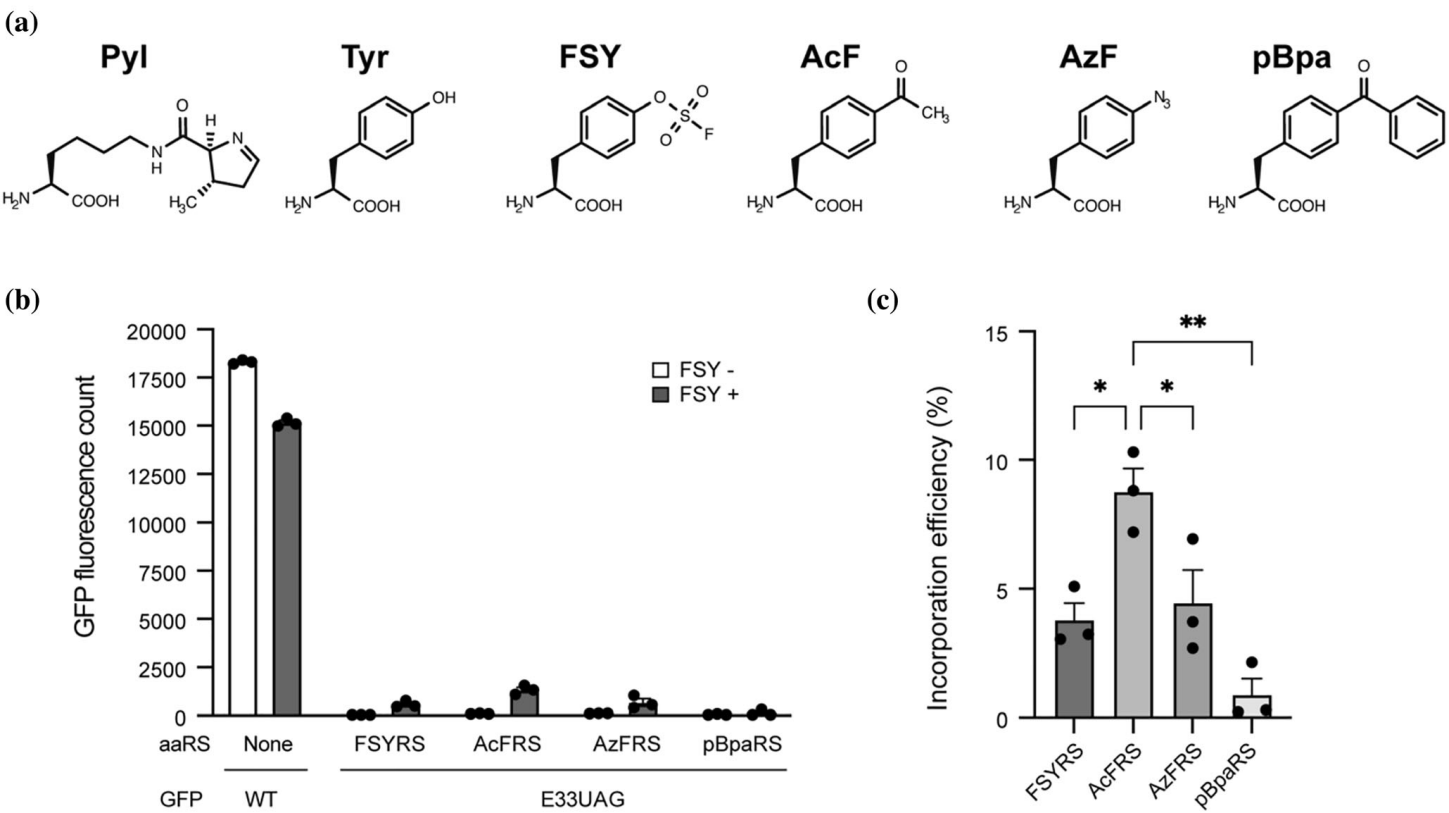
Site‐specific incorporation of FSY into EGFP with a series of aaRS variants. (a) Structures of L‐pyrrolysine (Pyl), L‐tyrosine (Tyr), p‐fluorosulfate‐L‐tyrosine (FSY), p‐acetyl‐L‐phenylalanine (AcF), p‐azide‐L‐phenylalanine (AzF), and p‐benzoyl‐L‐phenylalanine (pBpa). (b), (c) Expression of the full‐length EGFP(E33UAG) in 293 c 18 cells dependent on the indicated aaRSs and their cognate amber suppressor tRNAs in the presence and absence of 1 mM of FSY (*n* = 3). FSYRS indicates previously reported *M. mazei* PylRS variant specific for FSY. AcFRS, AzFRS, and pBpaRS indicate *Escherichia coli* TyrRS variants originally developed to recognize AcF, AzF, and pBpa, respectively. (b) Total fluorescence counts from the lysates of the cells. The values represent mean ± SE (*n* = 3). (c) Incorporation efficiencies of FSY at the UAG codon of EGFP represented by the percentages of EGFP fluorescence counts for each FSY+ sample in (b), as compared with that of the wild‐type EGFP. The values represent mean ± SE (*n* = 3). *p <0.05, ***p <0.001, *p*‐values were calculated with a one‐way ANOVA and Tukey–Kramer test.

FSY (Figure 1a) has previously been site‐specifically incorporated into proteins using orthogonal pairs of *Methanosarcina mazei* PylRS (*Mm*PylRS) variants and their cognate tRNA (Han et al., 2022; Wang et al., 2018; Yu et al., 2022). However, the reported incorporation efficiency of FSY using these PylRS‐based systems is relatively low compared to other ncAAs, potentially limiting protein yield (Jewel et al., 2024). This reduced efficiency may result from structural differences between FSY and pyrrolysine, the natural substrate for PylRS. Given that FSY is a para‐substituted phenylalanine derivative (Figure 1a), we explored whether *Escherichia coli* TyrRS (*Ec*TyrRS) variants, previously used to incorporate other para‐substituted phenylalanine analogs such as *p*‐acetyl‐L‐phenylalanine (AcF), *p*‐azido‐L‐phenylalanine (AzF), and *p*‐benzoyl‐L‐phenylalanine (pBpa) in eukaryotic cells (Chin et al., 2003; Hino et al., 2005, 2011; Liu et al., 2007), could also recognize and activate FSY. We co‐expressed several *Ec*TyrRS variants (AcFRS for AcF; AzFRS for AzF; pBpaRS for pBpa) or a previously reported FSY‐specific *Mm*PylRS variant (Wang et al., 2018) along with a compatible orthogonal amber suppressor tRNA (*Geobacillus stearothermophilus* tRNA^Tyr^_CUA_ for *Ec*TyrRS variants and *Mm*tRNA^Pyl^_CUA_ for *Mm*PylRS) and an enhanced green fluorescent protein (EGFP) reporter containing an amber stop codon at position 33 (EGFP[E33UAG]) in 293c18 cells. FSY was added to the culture medium at 1 mM. Successful FSY incorporation at the UAG codon enables expression of full‐length, fluorescent EGFP. We assessed FSY incorporation by measuring EGFP fluorescence intensity in cell lysates (Figure 1b). Significant fluorescence was observed in the presence of FSY for FSYRS, AcFRS, and AzFRS, with the highest signal obtained using AcFRS, yielding approximately twice the fluorescence of FSYRS (Figure 1b). We further estimated incorporation efficiency by comparing fluorescence intensities to those of WT EGFP expressed under similar conditions (Figure 1c). The AcFRS/tRNA^Tyr^_CUA_ pair achieved an incorporation efficiency of 8.8% ± 1.3%, significantly higher than the 3.8% ± 0.9% obtained with the FSYRS/tRNA^Pyl^_CUA_ pair (*p* <0.05). Based on its superior performance, we selected the AcFRS/tRNA^Tyr^_CUA_ system for all subsequent experiments involving FSY incorporation into ACE2‐Fc.

### 2.2 Designing the FSY incorporation site in ACE2‐Fc to target conserved nucleophilic residues on the RBD

**FIGURE 2:**
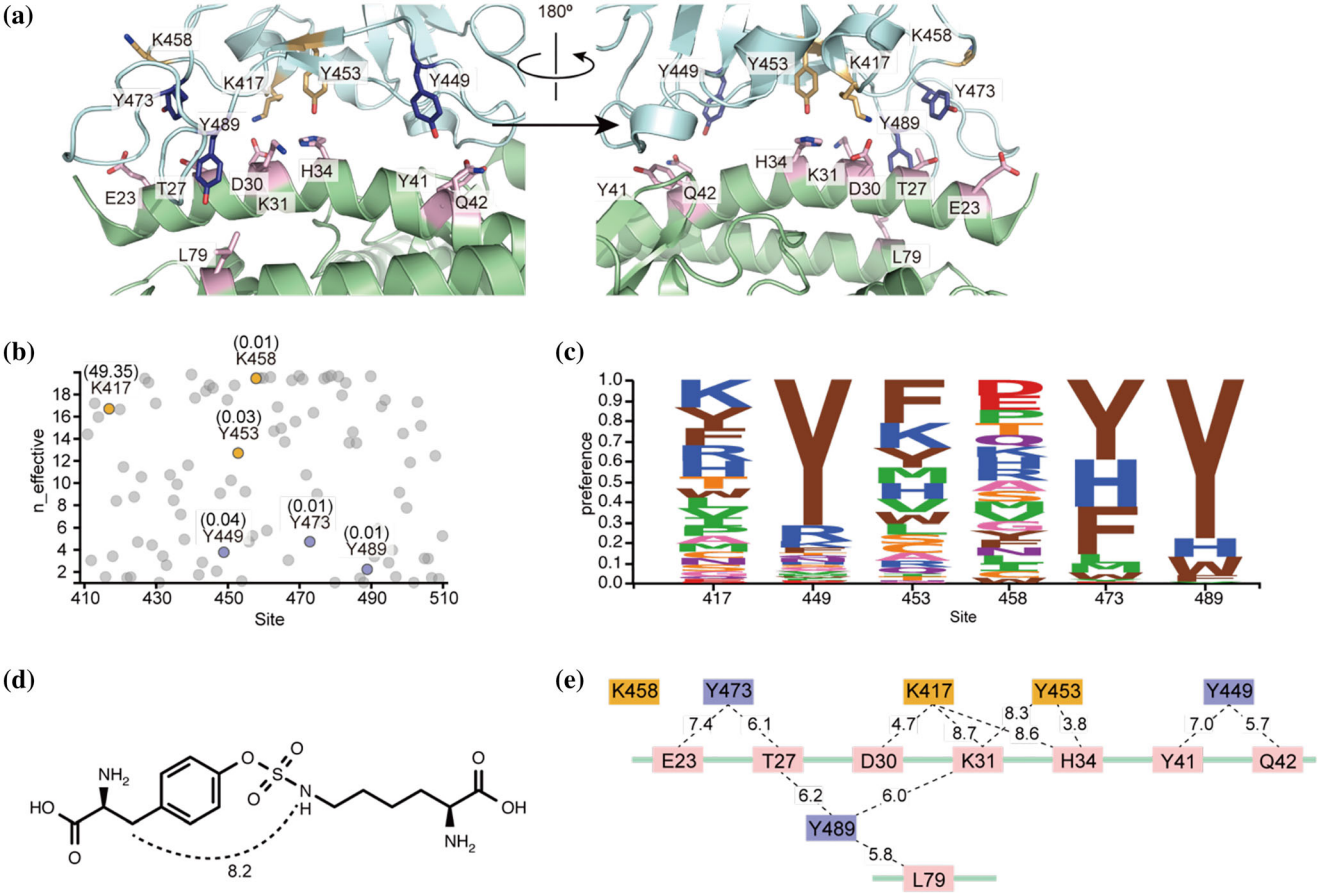
Design of FSY‐incorporation sites into ACE2‐Fc. (a) 3D structure of the binding interface between ACE2 (green) and RBD (cyan) (PDB ID: 7KMB). The candidate FSY‐incorporation sites on the ACE2 surface are shown in pink. The potential target residues on the RBD for covalent bonding with FSY are colored in orange and purple in accordance with the n‐effective values shown in (b). (b) Mutational tolerance of amino acid residues on the RBD for binding to ACE2 obtained from DMS analysis data (Starr et al., 2020) shown as *n*‐effective values, where lower values indicate stronger evolutionary constraint. The potential targeting residues are indicated and shown in orange (with higher *n*‐effective values) and in purple (with lower *n*‐effective values). The mutation frequencies in the real world from GISAID are also shown in parentheses. (c) Logoplots representing preferred amino acids at the targeted positions in the DMS analysis. (d) Chemical structure of a representative product from the SuFEx reaction of FSY with a nucleophilic residue (in this case, a lysine residue). The distance between the *β*‐carbon of FSY and the reacted nucleophilic atom is postulated to be within approximately 8.2 Å. (e) The distances between the *β*‐carbon of the ACE2 amino acids to be replaced with FSY and the nucleophilic atom in the side chain of potential targeting residues on the RBD calculated from the crystal structure shown in (a).

FSY specifically forms covalent bonds with nearby nucleophilic amino acid residues, including histidine, lysine, and tyrosine (Wang et al., 2018). To develop FSY‐incorporated ACE2‐Fc mutants capable of covalently capturing a broad spectrum of SARS‐CoV‐2 strains, it is essential to target nucleophilic residues on the RBD that are functionally constrained and therefore unlikely to mutate. Using the three‐dimensional (3D) structure of the ACE2–RBD complex (Figure 2a, Protein Data Bank [PDB] ID: 7KMB) (Zhou et al., 2020), we initially identified five candidate nucleophilic residues on the RBD: K417, Y449, Y453, Y473, and Y489. These residues possess reactive side chains and are located in proximity to the ACE2 binding interface. In addition, in silico simulation studies suggested that K458 of the RBD could form a salt bridge with E23 of ACE2 (Ali et al., 2021). K458 was therefore included, yielding a total of six RBD target residues.

We evaluated the mutational constraint of each residue using two metrics (Figure 2b). First, we assessed functional constraint using data from deep mutation scanning (DMS) (Starr et al., 2020; Starr et al., 2022), represented by the effective number of amino acids (*n*‐effective value). This value ranges from 1 (highly conserved) to 20 (fully tolerant to substitution). Lower values indicate stronger selection against mutations that disrupt ACE2 binding. As shown in Figure 2b, residues Y449, Y473, and Y489 had the lowest n‐effective values, suggesting stronger functional constraints than K417, Y453, and K458. Furthermore, logoplots derived from the DMS data illustrate the preferred amino acids at these positions (Figure 2c), highlighting the strong preference for tyrosine, particularly at positions 449, 473, and 489. Second, we evaluated the evolutionary conservation by analyzing the mutation frequencies of these residues in circulating SARS‐CoV‐2 virus sequences deposited in the Global Initiative on Sharing Avian Influenza Data database (n.d.) (GISAID accessed July 1, 2025). Consistent with the DMS results, mutation frequencies were extremely low (<0.05%) for Y449, Y473, and Y489. This level of conservation was also observed for Y453 and K458. In contrast, K417 showed a high mutation frequency (49.35%), mainly due to the prevalence of variants such as Beta and Omicron harboring the K417N mutation. Based on both high functional constraint and low mutation frequency, Y449, Y473, and Y489 were selected as optimal targets for broad‐spectrum neutralization.

We then identified suitable FSY incorporation sites on the ACE2‐Fc. The SuFEx reaction between FSY and a nucleophilic target residue is predicted to occur efficiently when the distance between the *β*‐carbon of FSY and the nucleophilic atom (nitrogen or oxygen) is within approximately 8.2 Å (Figure 2d). Using the ACE2–RBD complex structure (Figure 2a), we searched for ACE2 residues whose *β*‐carbons are oriented toward and within 9 Å of the nucleophilic atoms of our target residues (Y449, Y473, Y489), as well as the other candidate RBD residues. This slightly larger 9 Å cutoff accounts for potential protein structural fluctuations in solution. This analysis identified eight ACE2 residues—E23, T27, D30, K31, H34, Y41, Q42, and L79—as potential FSY incorporation sites (Figure 2a, pink). The distances between the β‐carbon of these ACE2 residues and the relevant nucleophile on the RBD are summarized in Figure 2e.

### 2.3 Cross‐linking of FSY‐incorporated ACE2‐Fc with the SARS‐CoV‐2 S protein

**FIGURE 3:**
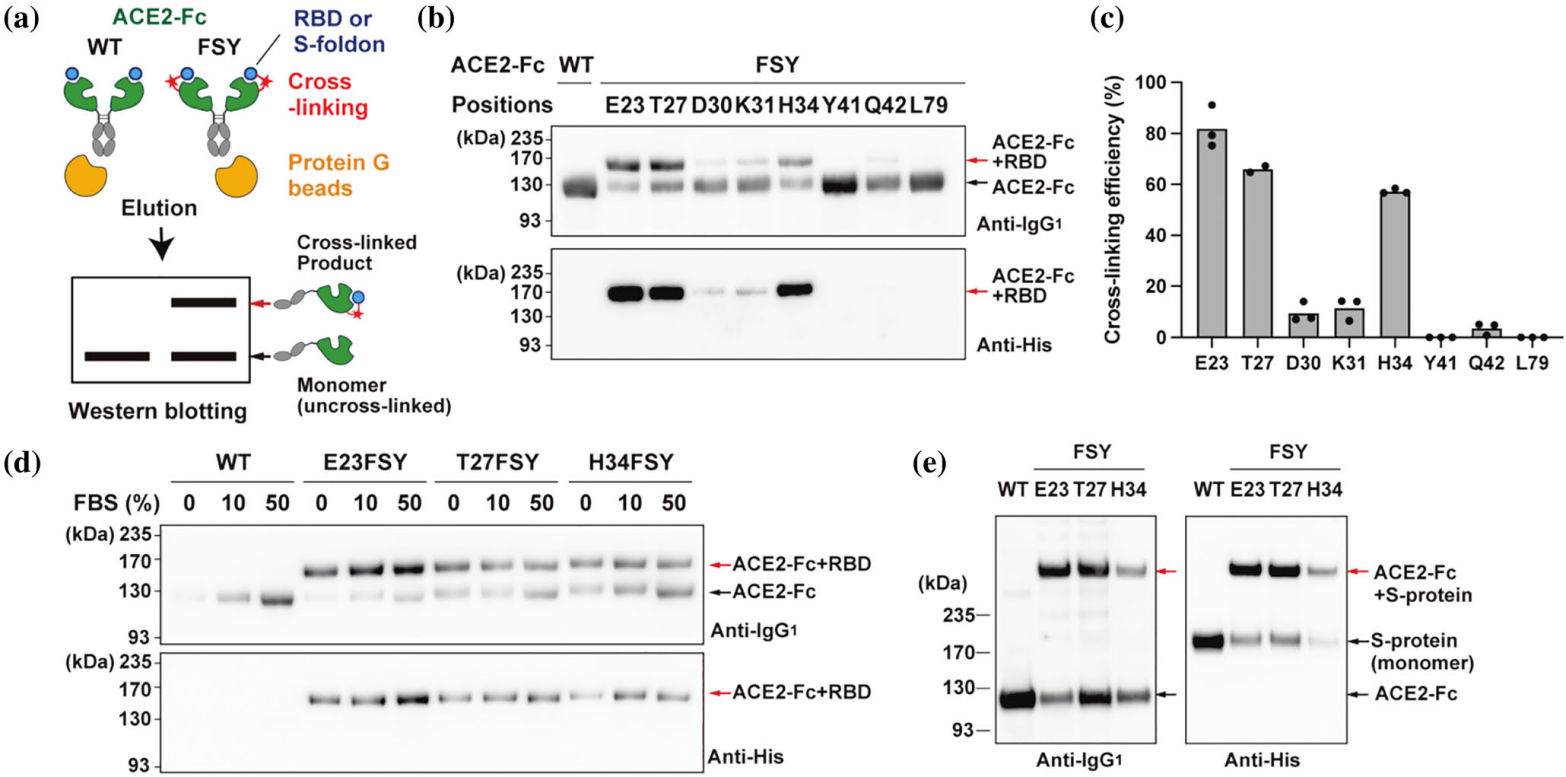
Cross‐linking between FSY‐incorporated ACE2‐Fc mutants and SARS‐CoV‐2 Spike proteins. (a) Schematic presentation of in vitro protein cross‐linking and western blot analysis. Cross‐linked product is detected as a shifted band on the blot. (b) On beads cross‐linking of ACE2‐Fc mutants containing FSY at the indicated positions with RBD‐His. The ACE2‐Fc mutants expressed in the culture supernatant of 293 c18 cells were trapped on Protein G beads and incubated with 1 μM of RBD‐His at 37°C for 20 h. Eluted proteins were analyzed by western blotting with an anti‐human IgG_1_ (top panel) and an anti‐His tag (bottom panel) antibody. ACE2‐Fc monomers and cross‐linked products are indicated with black and red arrows, respectively. (c) Cross‐linking efficiencies of the FSY‐incorporated ACE2‐Fc. Efficiencies were calculated by dividing the signal intensities of the cross‐linked product (ACE2‐Fc + RBD) by the sum of intensities of the cross‐linked product and the monomeric ACE2‐Fc band, based on the anti‐human IgG1 blot (*n* = 3, except for T27 sample [*n* = 2]). (d) In solution cross‐linking of ACE2‐Fc with RBD‐His in the presence of FBS. The indicated ACE2‐Fc constructs were expressed in cell culture supernatant. The supernatant was incubated with 0.25 μM of RBD‐His at 37°C for 20 h in the presence of FBS at the indicated concentrations (e.g., 10% or 50%). ACE2‐Fc proteins and their cross‐linked products were captured with Protein G beads and analyzed by western blotting. (e) On beads cross‐linking of ACE2‐Fc with trimerized ectodomain of S protein (S‐foldon). Cross‐linking was performed by incubating indicated ACE2‐Fc constructs and 0.4 μM of S‐foldon at 37°C for 20 h and analyzed by western blotting. The representative western blot images are shown.

To investigate the ability of FSY‐incorporated ACE2‐Fc mutants to covalently capture the RBD of SARS‐CoV‐2, 293c18 cells were transfected with plasmids encoding the AcFRS/tRNA^Tyr^_CUA_ pair and ACE2‐Fc mutants containing an amber codon at positions E23, T27, D30, K31, H34, Y41, Q42, or L79. The expressed ACE2‐Fc mutants were secreted into culture supernatant containing 1 mM FSY. A schematic overview of the representative in vitro cross‐linking assay is presented in Figure 3a. Briefly, the protein G beads coated with ACE2‐Fc were incubated with at least a 1.5‐fold molar excess of His‐tagged RBD (RBD‐His) at 37°C for 20 h in phosphate‐buffered saline (PBS). The proteins were then eluted and analyzed by western blot using both anti‐human IgG_1_ and anti‐His tag antibodies (Figure 3b). As shown in Figure 3b, all constructs, including WT ACE2‐Fc, produced a band at approximately 120 kDa, corresponding to the ACE2‐Fc monomer. In contrast, mutants with FSY at positions E23, T27, D30, K31, H34, and Q42 showed an additional, slower‐migrating band at approximately 160 kDa. This band was also detected with the anti‐His antibody (Figure 3b, bottom panel), confirming the covalent cross‐linking product with the approximately 35 kDa RBD‐His. These results demonstrate that FSY incorporated at these ACE2 sites mediates specific covalent bond formation with RBD. The highest cross‐linking efficiencies were observed for E23FSY (82%), T27FSY (66%), and H34FSY (57%). These three constructs were selected for further characterization.

To evaluate specificity and efficiency in more biologically relevant conditions, RBD‐His was added directly to culture supernatants containing the selected ACE2‐Fc mutants in the presence of 10% or 50% fetal bovine serum (FBS). Following protein G capture and western blotting, strong cross‐linking was observed in both serum conditions without notable non‐specific interactions with serum proteins, supporting the specificity of the SuFEx reaction (Figure 3d).

Finally, the assessment of cross‐linking with the full‐length trimeric SARS‐CoV‐2 S protein was conducted. A soluble, stabilized ectodomain of the D614G S variant (residues 1–1213), fused to a T4 foldon trimerization domain and a His tag, referred to as S‐foldon (Kawai et al., 2023), was used. The ACE2‐Fc bound beads were incubated with at least 1.5 molar excess of S‐foldon in PBS, and products were analyzed by sodium dodecyl sulfate–polyacrylamide gel electrophoresis (SDS‐PAGE) under denaturing conditions, followed by western blot. As shown in Figure 3e, FSY‐containing constructs produced a distinct high‐molecular weight band (>235 kDa) in addition to the monomeric ACE2‐Fc band (approximately 120 kDa). This upper band corresponds to a 1:1 covalent complex of ACE2‐Fc (approximately 120 kDa) and a monomer of S protein (approximately 180 kDa), likely observed after trimer dissociation during SDS‐PAGE. These results confirm that FSY‐mediated cross‐linking is effective even when RBD is presented in the context of the full trimeric S protein.

### 2.4 E23FSY and T27FSY mutants covalently capture the Omicron RBD by targeting highly conserved Y473

**FIGURE 4:**
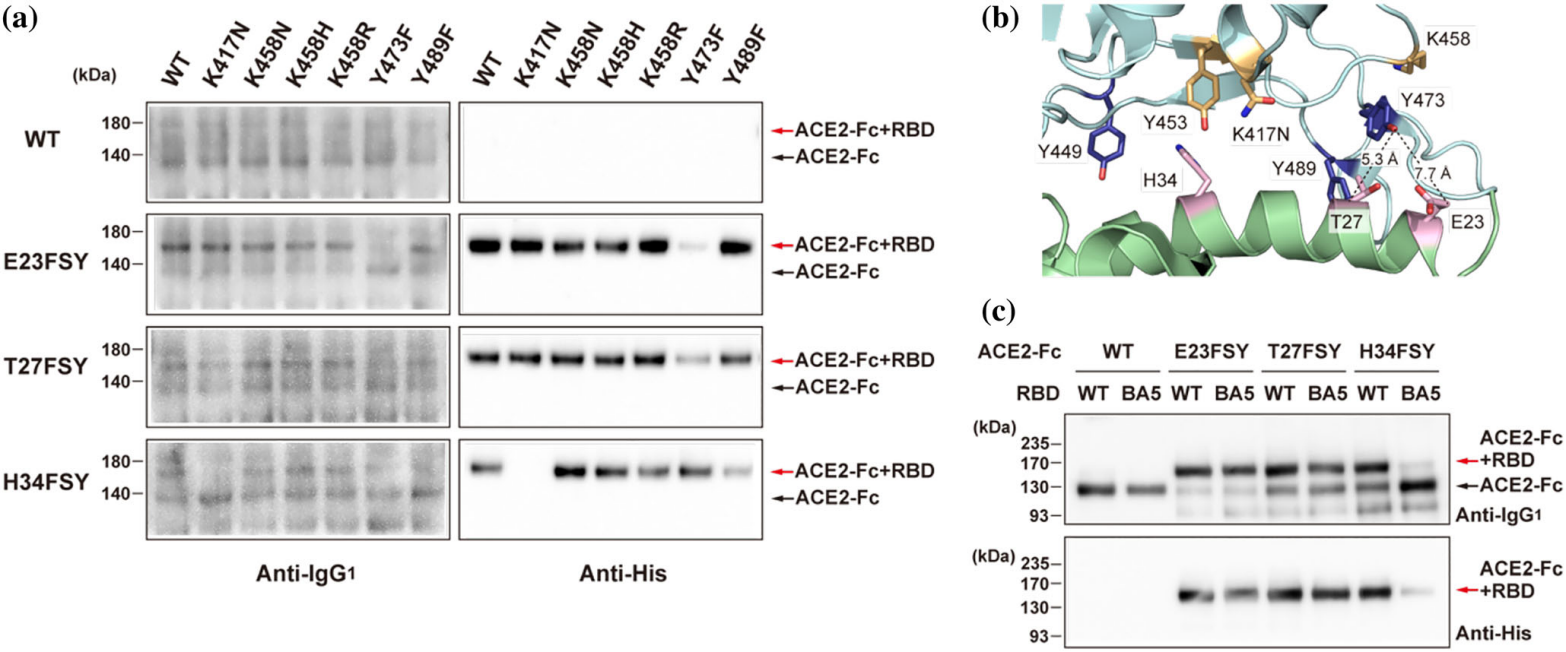
Cross‐linking between FSY‐incorporated ACE2‐Fc mutants and RBD variants. (a) Determining the target residues on RBD for ACE2‐Fc constructs. Cell supernatants containing indicated ACE2‐Fc mutants were incubated with 0.04 μM (at least 10 molar excess of ACE2‐Fc constructs) of various RBD‐His point mutants at 37°C for 20 h. The samples were directly analyzed by western blot analysis. (b) 3D structure of the binding interface between ACE2 and Omicron BA.5 RBD (PDB ID: 7WPB) colored as in Figure 2a. (c) Comparison of cross‐linking of ACE2‐Fc constructs with WT and Omicron BA.5 RBDs. Indicated ACE2‐Fc constructs were incubated with 0.04 μM (at least 10 molar excess of ACE2‐Fc constructs) of WT or Omicron BA.5 RBD‐His under conditions similar to (a). Subsequent capture with Protein G beads and western blot analysis was performed as described in Figure 3d. The experiments were conducted at least twice and representative western blot images are shown.

Next, we sought to determine the specific residues on the RBD targeted by FSY incorporated at positions E23, T27, and H34 of the ACE2‐Fc. To this end, we prepared a panel of RBD‐His point mutants designed to eliminate potential nucleophilic targets, including K417N and variants at position 458 (K458N, K458R, K458H), as well as Y473F and Y489F. These RBD variants, along with WT RBD, were incubated with ACE2‐Fc constructs, and cross‐linking was assessed by western blot (Figure 4a). The results revealed distinct target specificities: both E23FSY and T27FSY effectively cross‐linked with WT RBD and all tested point mutants except Y473F, where cross‐linking was markedly reduced. Conversely, H34FSY cross‐linked with WT and all mutants except K417N, indicating loss of reactivity (Figure 4a). These findings strongly suggest that E23FSY and T27FSY target Y473 on the RBD, while H34FSY targets K417. This target specificity aligns with structural modeling predictions based on residue proximity (Figure 2e).

It is well established that RBDs from highly mutated Omicron subvariants frequently carry mutations at position K417 (such as K417N), whereas the Y473 residue remains highly conserved across nearly all SARS‐CoV‐2 variants, including Omicron lineages (Cao et al., 2022; GISAID accessed July 1, 2025; Shrestha et al., 2022). To structurally confirm the feasibility of targeting Y473 in Omicron variants, we examined the 3D complex structure of the Omicron BA.5 RBD and ACE2 (Figure 4b, PDB ID: 7WPB) (Yin et al., 2022). The analysis revealed that Y473 remains close to E23 and T27 on ACE2, with distances from the nucleophilic oxygen atom (Oη) of Y473 to the *β*‐carbons of E23 and T27 calculated as 7.7 and 5.3 Å, respectively (Figure 4b).

These structural evidences, combined with the high conservation of Y473, supported our hypothesis that E23FSY and T27FSY ACE2‐Fc mutants could effectively capture the Omicron BA.5 RBD. Indeed, western blot analysis comparing cross‐linking with WT and Omicron BA.5 RBD (Figure 4c) demonstrated that both E23FSY and T27FSY, but not H34FSY, robustly cross‐linked to the Omicron BA.5 RBD with efficiencies comparable to those observed for the WT RBD. These results confirm the target specificity identified using single‐point mutants (Figure 4a) and underscore the advantage of targeting conserved residues like Y473 for broad‐spectrum variant neutralization.

### 2.5 Kinetics of the cross‐linking reaction between FSY‐containing ACE2‐Fc and RBD

**FIGURE 5:**
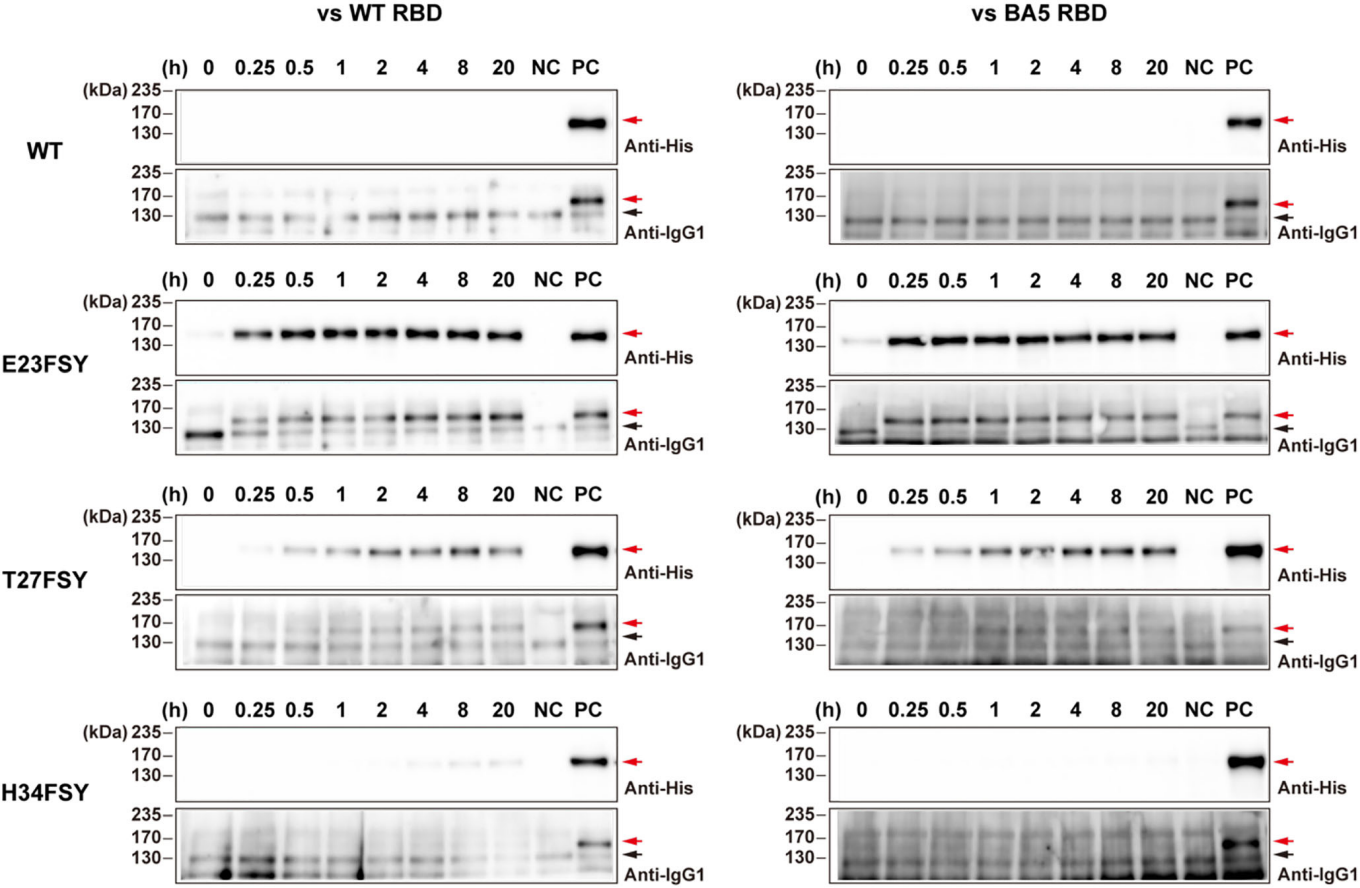
Time course analysis of covalent cross‐linking between ACE2‐Fc constructs and WT or Omicron BA.5 RBD. Cell culture supernatants containing indicated ACE2‐Fc constructs (WT, E23FSY, T27FSY, H34FSY), at an estimated concentration of 2.5 nM each, were incubated with 25 nM of either WT RBD‐His (left panels) or Omicron BA.5 RBD‐His (right panels) at 37°C for indicated time points. The samples were directly analyzed by western blotting. NC (Negative Control): WT ACE2‐Fc incubated with the respective RBD for 20 h at 37°C. PC (Positive Control): ACE2‐Fc (E23FSY) incubated with the respective RBD for 20 h at 37°C. The experiments were conducted twice, and representative western blot images are shown.

To quantitatively evaluate the rate at which ACE2‐Fc constructs containing FSY form covalent bonds with the RBD of WT and Omicron BA.5 RBD variants of SARS‐CoV‐2, a time‐course analysis of the cross‐linking reaction was performed (Figure 5). Cell culture supernatants containing the respective ACE2‐Fc constructs (approximate concentration 2.5 nM or 0.3 ng/μL) were incubated with 25 nM RBD‐His at 37°C for time points ranging from 0 to 20 h. The monomeric WT ACE2‐Fc remained stable over the full incubation period, indicating no covalent reaction. In contrast, FSY‐containing mutants showed a time‐dependent decrease in monomer and a corresponding increase in cross‐linked product. For WT RBD, E23FSY and T27FSY achieved near‐maximal cross‐linking within 0.5–2 h, while H34FSY reacted significantly slower, with detectable cross‐linked product only after 4 h. Reactivity was even higher with Omicron BA.5 RBD, with E23FSY and T27FSY reaching maximum yield within 0.25–1 h. H34FSY, however, failed to form detectable cross‐linked products with Omicron BA.5 RBD over the entire 20‐h incubation period, consistent with prior findings (Figure 4c).

### 2.6 Neutralization of viral infection

**FIGURE 6:**
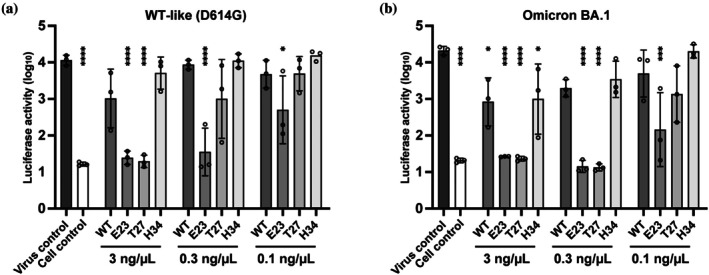
Neutralization of pseudo‐typed SARS‐CoV‐2 viruses by FSY‐incorporated ACE2‐Fc mutants. Pseudo‐typed WT‐like (D614G) (a) and Omicron BA. 1 (b) SARS‐CoV‐2 entry into VeroE6/TIMPRSS2 cells. The viruses were pre‐incubated with 3, 0.3, and 0.1 ng/μL of WT and FSY‐incorporated ACE2‐Fc constructs or PBS (Virus control) at 37°C for 2 h, and added to the cell culture medium of the cells. Luciferase activity was measured 48 h post infection. Cell control indicates the sample without viral infection. Data are expressed as the log_10_ mean ± SE of log_10_ mean (*n* = 3). *p <0.05, ***p <0.001, ****p <0.0001, *p*‐values were calculated with a one‐way ANOVA and Dunnett's test, versus virus control.

To assess the neutralizing capacity of the lead covalent ACE2‐Fc constructs (E23FSY and T27FSY), we evaluated their ability to block SARS‐CoV‐2 entry, using a control covalent construct (H34FSY) and non‐covalent WT ACE2‐Fc for comparison. Neutralization activity was assessed using pseudotyped vesicular stomatitis virus particles bearing either the D614G (WT‐like) or Omicron BA.1 S protein and carrying a luciferase reporter gene (Kawai et al., 2023). Similar to the Omicron BA.5 spike, the BA.1 spike contains the K417N mutation and retains the conserved Y473 residue (Shrestha et al., 2022). Pseudoviruses were pre‐incubated with varying concentrations of each ACE2‐Fc construct for 2 h before being added to VeroE6 cells expressing transmembrane protease serine 2 (TMPRSS2). Viral entry was quantified by measuring luciferase activity (Figure 6). Against the WT‐like D614G pseudovirus (Figure 6a), E23FSY showed potent inhibition at the lowest tested dose (0.1 ng/μL), while T27FSY was effective at 3.0 ng/μL. In contrast, both the non‐covalent WT ACE2‐Fc and the H34FSY mutant lacked significant neutralizing activity. The failure of the H34FSY mutant to neutralize viruses is probably due to its slow cross‐linking kinetics (Figure 5). Importantly, this enhanced neutralizing effect was retained against the Omicron BA.1 pseudovirus (Figure 6b). E23FSY and T27FSY significantly reduced viral entry at 0.1 and 0.3 ng/μL, respectively. WT ACE2‐Fc showed weak activity only at the highest concentration (3.0 ng/μL), while H34FSY again failed to neutralize the virus. This is consistent with its inability to covalently bind the K417N‐containing Omicron RBD (Figure 4).

These results demonstrate that the rationally designed covalent ACE2‐Fc constructs, E23FSY and T27FSY, confer dramatically enhanced and broad neutralizing activity against both WT‐like and Omicron SARS‐CoV‐2 variants. This underscores the value of targeting conserved and mutation‐constrained residues such as Y473, compared to non‐covalent ACE2‐Fc and a covalent construct targeting a mutation‐prone RBD residue. These results highlight that designing FSY incorporation sites to engage mutation‐constrained amino acid residues on the RBD is a highly effective strategy for creating broad and potent viral neutralizers.

## 3 DISCUSSION

In this study, we successfully developed a covalent ACE2‐Fc decoy receptor capable of potently neutralizing multiple variants of SARS‐CoV‐2. Using a rational design strategy informed by functional genomics and structural data, we identified three highly conserved residues on the viral RBD—Y449, Y473, and Y489—as optimal targets for covalent interaction. Among the designed constructs, the ACE2‐Fc mutants E23FSY and T27FSY, which target Y473, demonstrated rapid and specific cross‐linking to the RBD. These constructs exhibited significantly enhanced neutralization activity against both a WT‐like SARS‐CoV‐2 pseudovirus and the heavily mutated Omicron BA.1 variant.

Our findings emphasize the importance of strategic residue selection in antiviral design. A prior study targeting K417 (Yu et al., 2022) demonstrated vulnerability to the K417N mutation found in Beta and Omicron variants, which abolished covalent binding capacity. In contrast, we leveraged deep mutational scanning (Starr et al., 2020) and genomic surveillance data (GISAID accessed July 1, 2025) to select Y473, a residue that is functionally important and conserved across circulating lineages. As shown in Figure 4, constructs targeting Y473 successfully formed covalent bonds with even the Omicron BA.5 RBD. The enhanced neutralization observed in our constructs (Figure 6) likely results from the irreversible SuFEx reaction mediated by FSY. Unlike reversible binders, including high‐affinity engineered ACE2 decoys (Arimori et al., 2022), which maintain a dynamic binding‐dissociation equilibrium, FSY‐modified constructs form permanent covalent bonds with the viral S protein upon interaction. This irreversible binding, particularly with the rapid kinetics observed for E23FSY (Figure 5), leads to efficient viral inactivation.

Recently, the strategy of creating covalent binders using FSY has been successfully applied to protein nanobodies and minibinders against SARS‐CoV‐2, demonstrating enhanced neutralization efficacy against variants (Han et al., 2022; Yu et al., 2022). Although these studies powerfully validate the potential of covalent modification, our work highlights the distinct advantages of using the native receptor, ACE2, as the engineering platform. A primary advantage lies in the design process itself. The rational design of covalent binders is critically dependent on high‐resolution structural information. For novel nanobodies and minibinders, this often requires a preliminary, resource‐intensive step of determining the co‐crystal structure of each new binder with the RBD. In contrast, our approach leverages the well‐characterized, publicly available structure of the native ACE2‐RBD complex, allowing for a more direct and reliable structure‐guided design of FSY incorporation sites. Furthermore, the nature of the interaction interface offers a fundamental advantage. Nanobodies typically recognize relatively small, specific epitopes on the RBD surface, often imperfectly masking the ACE2 binding interface (Ahmad et al., 2021; Pymm et al., 2021). In contrast, the natural interaction between ACE2 and the RBD occurs across a broad, continuous surface area. This expansive interface inherently presents a larger number of potential nucleophilic residues (His, Lys, Tyr) within a suitable distance for covalent targeting. Consequently, the ACE2‐Fc platform provides a wider “design space,” increasing the probability of identifying an optimal FSY incorporation site that can target a residue like Y473, which satisfies the dual criteria of being highly conserved and sterically accessible for the SuFEx reaction. Recently, Meng et al. developed a covalently engineered ACE2 vesicle derived from the 293T cell membrane expressing ACE2 with FSY at position E23 (Meng et al., 2025). The nanovesicle was shown to covalently capture WT and Omicron RBDs, consistent with our data using ACE2 (E23FSY)‐Fc, and efficiently inhibited infection by WT and Omicron SARS‐CoV‐2, reducing animal mortality when used as an inhalable spray. Nevertheless, our design and validation strategy of incorporating FSY into ACE2‐Fc to target the mutation‐constrained residues of RBD remains valuable for the rational development of broad‐spectrum covalent antiviral therapeutics.

This study has some limitations. First, as we conducted neutralization assays using pseudoviruses, the efficacy of the covalent decoys will need to be validated against authentic SARS‐CoV‐2, including currently circulating variants, in both in vitro and in vivo models. Second, because the FSY‐incorporated ACE2‐Fc must be expressed in mammalian cells such as 293c18, the protein yield was quite low (sub‐microgram levels per 10‐cm dish), compared to nanobodies reportedly produced in bacterial cultures at 1–2.5 mg/L (Yu et al., 2022). This highlights the need for a reliable method to generate stable mammalian cell lines capable of producing high yields of proteins containing ncAAs such as FSY (Roy et al., 2020). Finally, although Y473 is highly conserved across SARS‐CoV‐2 variants, the corresponding residue is a phenylalanine in SARS‐CoV and is absent in some other sarbecoviruses (Hu et al., 2021; V'Kovski et al., 2021). This suggests that, while our strategy is robust against the intra‐species evolution of SARS‐CoV‐2, its broader applicability to the sarbecoviruses family will require careful selection of target residues that are conserved across multiple species.

In conclusion, this work establishes a rational framework for designing broad‐spectrum covalent viral neutralizers. By integrating functional genomics data with structural engineering on a native receptor scaffold, we created promising therapeutic candidates with robust activity against SARS‐CoV‐2 and its evolving variants. Covalent decoy engineering represents a powerful and widely available approach with potential applications for future viral pandemics.

## 4 MATERIALS AND METHODS

### 4.1 Plasmids

The pOriP vector that contains the replication origin of Epstein–Barr virus was used for high‐level gene expression in 293c18 cells (Mukai et al., 2008). pOriP‐based vectors carrying the genes for WT and amber mutant (E33UAG) EGFP, *Mm*PylRS, and nine copies of *Mm*tRNA^Pyl^_CUA_ were described previously (Kita et al., 2016; Mukai et al., 2008). The pOriP‐based pSuPT vectors carrying genes for nine copies of *Gs*tRNA^Tyr^_CUA_ and *Ec*TyrRS‐derived aminoacyl‐tRNA synthetases AcFRS, AzFRS, and pBpaRS were also described previously (Hino et al., 2011). pSuPC vectors expressing pairs of *Gs*tRNATyr and *Ec*TyrRS variants were created by replacing the thymidine kinase promoter of the pSuPT vectors with a cytomegalovirus (CMV) promoter. The gene fragment encoding the C‐terminal region (amino acids 246–454) of *Mm*PylRS variant specific to FSY (i.e., 302I/346T/348I/384L/417K variant) (Wang et al., 2018) was chemically synthesized, amplified by polymerase chain reaction (PCR), and used to replace the corresponding region of the WT *Mm*PylRS gene on the pOriP vector using the In‐Fusion HD Cloning Kit (Takara Bio Inc., Shiga, Japan) to generate the expression vector for FSYRS (pOriP‐FSYRS). The gene for ACE2‐Fc, which encodes a fusion protein comprising the ectodomain (amino acids 1–615) of human ACE2 and the Fc region of human IgG_1_, was amplified from pcDNA3‐sACE2(WT)‐Fc(IgG_1_) (a gift from Erik Procko; Addgene plasmid # 145163) and cloned downstream of the CMV promoter in pOriP to generate pOriP‐ACE2‐Fc. pOriP‐ACE2(740)‐Fc was constructed by inserting the C‐terminal region of the ectodomain (amino acids 601–740) amplified from the 3N39v3 ACE2‐Fc decoy‐encoding vector (Ikemura et al., 2022), between the ACE2 (amino acids 1–600) and Fc coding region of pOriP‐ACE2‐Fc. pOriP‐ACE2(740)‐Fc‐His was generated by fusing a glycine‐serine linker and an 8x His‐tag sequence to the C‐terminus of the Fc fragment in pOriP‐ACE2(1–740)‐Fc via PCR using overhang primers. The gene fragment encoding the RBD with an N‐terminal secretion signal from human interleukin‐2 and a C‐terminal 8× His‐tag was amplified from pcDNA3.1‐RBD(WT)‐His and pcDNA3.1‐RBD(BA.5)‐His (Kawai et al., 2023), and cloned downstream of the CMV promoter in pOriP. Point mutations in ACE2‐Fc, ACE2(1–740)‐Fc‐His, and RBD‐His constructs were generated by site‐directed mutagenesis. The PCR primers and synthetic gene fragments used are listed in Supplementary Table 1.

### 4.2 Incorporation of FSY into EGFP in mammalian cells

Cells (293c18, American Type Culture Collection) were seeded at 2.0 × 10^5^ cells per well in a 24‐well plate pre‐coated with poly‐D‐lysine (BD) and cultured in Dulbecco's Modified Eagle's Medium (DMEM) high glucose supplemented with 10% (v/v) heat‐inactivated FBS (Sigma‐Aldrich, St. Louis, MO, USA) for 24 h at 37°C in 5% CO_2_. pOriP‐EGFP(E33UAG) was co‐transfected into the cells along with pOriP plasmids encoding FSYRS and *Mm*tRNA^Pyl^_CUA_, or with pSuPC plasmids encoding *Gs*tRNA^Tyr^_CUA_ and an *Ec*TyrRS variant (AcFRS, AzFRS, or pBpaRS) using Lipofectamine 2000 in Opti‐MEM I reduced‐serum medium (Thermo Fisher Scientific, Waltham, MA, USA), following the manufacturer's protocol. After a 6‐h incubation, the medium was replaced with DMEM high glucose without FBS but supplemented with 1 mM FSY (Enamine Kiev, Ukraine). After an additional 18‐h incubation, the cells were lysed in lysis buffer (50 mM Tris–HCl [pH 7.5], 150 mM NaCl, 5 mM ethylenediaminetetraacetic acid, 1% Triton X‐100, 10% glycerol, and protease inhibitor cocktail [Roche, Basel, Switzerland]) and transferred to a 96‐well black plate. EGFP fluorescence was measured at excitation/emission = 475/500–550 nm using a GloMax Discover microplate reader (Promega, Madison, WI, USA).

### 4.3 Expression and purification of His‐tagged RBD and S proteins

For small‐scale preparation, 293c18 cells were seeded at 3.2 × 10^6^ cells per 10‐cm dish and cultured for 24 h at 37°C with 5% CO_2_. Cells were then transfected with a series of pOriP plasmids encoding WT and the point mutants of the RBD with C‐terminal His‐tags using Lipofectamine 2000 in Opti‐MEM I. After 48 h of incubation, the culture supernatant was collected, filtered through a 0.45 μm pore filter (Millipore, Burlington, MA, USA), and concentrated 10‐fold using a Vivaspin 6 ultrafiltration unit (<10,000 Da cutoff, Sartorius, Göttingen, Germany). Next, 30 μL (bed volume: 1.5 μL) of His Mag Sepharose Ni beads (Cytiva) was added to the concentrated medium and incubated at 4°C for 1 h. The beads were washed twice with PBS containing 5 mM imidazole. Proteins bound to the beads were eluted with 100 μL of PBS containing 500 mM imidazole and filtered through Zeba Spin Desalting Columns (<7000 Da cutoff; Thermo Fischer Scientific) to remove the imidazole. A portion of the purified proteins was subjected to SDS‐PAGE followed by Coomassie Brilliant Blue (CBB) staining. Protein concentration was determined by densitometry using a bovine serum albumin standard curve and Image J software.

For large‐scale preparation, 7.5 × 10^7^ Expi293F cells in 30 mL of medium were transfected with pcDNA3.1 plasmids encoding RBD‐His or S‐foldon (Kawai et al., 2023) using ExpiFectamine 293 Reagent (Thermo Fisher Scientific). After 18 h of incubation at 37°C with 8% CO_2_ on an orbital shaker (120 rpm), ExpiFectamine 293 Transfection Enhancers 1 and 2 were added. The culture was further incubated, and the supernatant was collected. His‐tagged proteins were purified using an AKTA Explorer chromatography system equipped with a Ni‐Sepharose HisTrap FF column (Cytiva) and further purified via size‐exclusion chromatography on a Superose 6 Increase 10/300 GL column (Cytiva).

### 4.4 Expression and purification of ACE2‐Fc constructs

Cells (293c18) were seeded at 3.2 × 10^6^ cells in 10‐cm dishes and cultured in DMEM high glucose supplemented with 10% (v/v) heat‐inactivated FBS for 24 h at 37°C with 5% CO_2_. For the expression of FSY‐containing ACE2‐Fc, 3 μg of pOriP vectors encoding a UAG mutant of ACE2‐Fc were co‐transfected with 5 μg of pSuPC‐AcFRS using 20 μL of Lipofectamine 2000 in 5 mL of Opti‐MEM I. For the expression of WT ACE2‐Fc, 0.3 μg of pOriP‐ACE2‐Fc were co‐transfected with 2.7 μg of pOriP empty vector and 5 μg of pSuPC‐AcFRS to balance the expression with FSY‐containing mutants. After 6 h, FSY was added to the culture medium to a final concentration of 1 mM, followed by an additional 44‐h incubation. The culture supernatant was centrifuged, filtered through a polyvinylidene fluoride (PVDF) membrane, and concentrated approximately 10‐fold using an Amicon ultrafiltration unit (<100,000 Da cutoff, Millipore). For purification, the medium was incubated with Protein G Mag Sepharose beads (Cytiva, Marlborough, MA, USA) for 1 h at 4°C with rotation. After washing twice with PBS, proteins were eluted with 0.1 M glycine (pH 3.0), and the pH was neutralized with 1.5 M Tris–HCl (pH 8.8). The WT and FSY‐containing ACE2(740)‐Fc‐His proteins used for the pseudovirus neutralization assay were expressed in 2 mM FSY‐containing medium using 6 and 18 10‐cm dishes of 293c18 cells, respectively. The culture supernatant was centrifuged, filtered through a PVDF membrane, and concentrated to 1 mL using a Vivaspin Turbo 15RC ultrafiltration unit (<100,000 Da cutoff, Sartorius, Göttingen, Germany). For purification, the concentrated supernatant was incubated with 100 μL (bed volume: 5 μL) of His Mag Sepharose Ni beads at 4°C for 1 h. The beads were sequentially washed with PBS containing 5 mM and 50 mM imidazole, then incubated in PBS containing 200 mM imidazole at 25°C for 10 min to elute the proteins. The eluate was filtered through Zeba Spin Desalting Columns (<40,000 Da cutoff; Thermo Fischer Scientific) to remove imidazole. A portion of the purified proteins was subjected to SDS‐PAGE followed by CBB staining. Protein concentration was calculated as described above.

### 4.5 Cross‐linking

For on‐beads cross‐linking (Figure 3b,e), half of the culture supernatant from a 10‐cm dish was concentrated 10‐fold and trapped on the protein G beads as described above. The beads were mixed with either RBD‐His (final concentration, 1 μM) or S‐foldon (final concentration, 0.4 μM) and incubated for 20 h at 37°C. For in‐solution cross‐linking (Figures 3d, 4a,c), the concentrated supernatant containing ACE2‐Fc proteins was incubated with at least 10 molar excess of RBD‐His proteins with or without FBS for 20 h at 37°C. For the time course analysis (Figure 5), approximately 2.5 nM (0.3 ng/μL) of ACE2‐Fc proteins in Opti‐MEM I medium was incubated with 25 nM of RBD‐His proteins at 37°C, and aliquots were collected at the indicated time points. The ACE2‐Fc proteins and their cross‐linked products were purified with protein G beads (Figures 3d and 4c) as described above or used directly (Figures 4a and 5) for the following analysis. The proteins were separated by SDS‐PAGE and analyzed by western blotting with horse radish peroxidase‐conjugated anti‐human IgG_1_ and anti‐His antibodies (Proteintech, Rosemont, IL, USA) at 1:3000 and 1:10000 dilutions, respectively.

### 4.6 Pseudovirus neutralization assay

Pseudovirus neutralization assay was performed as described previously (Kawai et al., 2023), with some modifications. VeroE6/TMPRSS2 cells were seeded at 1.2 × 10^4^ cells per well in a 96‐well half‐white plate and incubated in the medium (DMEM high glucose, 10% [v/v] heat‐inactivated FBS, 1% [v/v] penicillin–streptomycin [FUJIFILM, Tokyo, Japan]) for 24 h at 37°C. On the day of infection, WT and FSY‐incorporated ACE2(740)‐Fc‐His constructs, diluted in 15 μL of medium to final concentrations of 6.0, 0.6, and 0.2 ng/μL, were incubated with 15 μL of WT‐like (D614G) or Omicron BA.1 pseudovirus solution for 2 h at 37°C. The mixture was diluted threefold with the medium. A volume of 50 μL of this mixture was added to the cells, replacing the growth medium, and incubated at 37°C for 48 h. After virus infection, 50 μL of One‐GloTM‐EX Reagent (Promega) was added, followed by chemiluminescence measurement on a microplate reader (Powerscan HT, DS Pharma Biomedical, Osaka, Japan).

### 4.7 Statistics

Statistical analyses were performed using Prism 10 (version 10.5.0) software (GraphPad Software Inc., San Diego, CA). Significant differences were determined using one‐way analysis of variance, followed by Tukey‐Kramer or Dunnett's test. *p* <0.05 was considered statistically significant.

## AUTHOR CONTRIBUTIONS

**Nobumasa Hino:** Conceptualization; funding acquisition; writing – original draft; supervision; formal analysis; project administration; visualization; writing – review and editing; data curation; validation. **Risa Takada:** Investigation; formal analysis; visualization; writing – original draft. **Kyosuke Suzuki:** Investigation; formal analysis; visualization; writing – original draft. **Nagisa Tokunoh:** Investigation; formal analysis; validation; visualization. **Tatsuya Karaki:** Investigation; validation. **Kazumasa Ohtake:** Funding acquisition; investigation; conceptualization. **Haruna Ogami:** Validation; investigation. **Takafumi Nishiura:** Investigation; validation. **Kenji Ishimoto:** Supervision; writing – review and editing. **Tomohito Tsukamoto:** Supervision; writing – review and editing. **Yukio Ago:** Supervision; writing – review and editing. **Yoshiaki Okada:** Supervision; writing – review and editing. **Toru Okamoto:** Resources; writing – review and editing. **Chikako Ono:** Resources; writing – review and editing. **Yoshiharu Matsuura:** Resources; writing – review and editing. **Satoshi Obika:** Supervision; writing – review and editing. **Kensaku Sakamoto:** Supervision; resources; writing – review and editing; funding acquisition; conceptualization. **Yasuo Yoshioka:** Conceptualization; data curation; supervision; project administration; writing – review and editing; funding acquisition. **Shinsaku Nakagawa:** Writing – review and editing; project administration; supervision; funding acquisition.

## CONFLICT OF INTEREST STATEMENT

The authors declare the following competing financial interest(s). N.T., T.K., and Y.Y. are employees of the Research Foundation for Microbial Diseases of Osaka University. The other authors declare no conflicts of interest.

## Supporting information


**Supplementary Table 1:** List of primers and synthetic gene fragments,
