# Supplementary material for: Rational design of a covalent ACE2 decoy receptor that broadly neutralizes SARS‐CoV‐2 variants
Source: Protein Sci. 2025 Sep 13;34(10):e70306. doi: 10.1002/pro.70306 (PMC12432405; doi:10.1002/pro.70306)
Supplement: Supplementary file 1 — Supplementary Table 1: List of primers and synthetic gene fragments, [file PRO-34-e70306-s001.docx]

Supplementary Table I List of primers and synthetic gene fragments.

| Name | Sequence (5'-3') | Usage |
| --- | --- | --- |
| 5_KpnI_ACE2-Fc | cacacaggtaccaccatgtcaagc | Subcloning |
| 3_XhoI_ACE2-Fc | cacacactcgagttatcatttacccgg | Subcloning |
| 5_EcoRI-ACE2(601-) | cacacagaattcttttgtgggatggagtaccg | Subcloning |
| 3_BamH1-ACE2(-740) | cacacaggatccggaaacagggggctggttag | Subcloning |
| 3_XhoI-Fc-GS-8xHis | cacacactcgagttatcaatgatgatggtggtggtgatggtgggatcctttacccggagacagggagagg | Subcloning |
| FSYRS Fragment FW | tctggggaaactcgagcgtgaaattaccaggttctt | Subcloning |
| FSYRS Fragment RV | gccctctagactcgagttacaggttggtagaaatcc | Subcloning |
| QC5_ACE2_E23UAG | ccaccattgagTAGcaggccaagac | Mutagenesis |
| QC3_ACE2_E23UAG | gtcttggcctgCTActcaatggtgg | Mutagenesis |
| QC5_ACE2_T27UAG | caggccaagTAGtttttggacaag | Mutagenesis |
| QC3_ACE2_T27UAG | cttgtccaaaaaCTActtggcctg | Mutagenesis |
| QC5_ACE2_D30UAG | ccaagacatttttgTaGaagtttaaccacg | Mutagenesis |
| QC3_ACE2_D30UAG | cgtggttaaacttCtAcaaaaatgtcttgg | Mutagenesis |
| QC5_ACE2_K31UAG | gacatttttggacTagtttaaccacg | Mutagenesis |
| QC3_ACE2_K31UAG | cgtggttaaactAgtccaaaaatgtc | Mutagenesis |
| QC5_ACE2_H34UAG | gacaagtttaacTaGgaagccgaagac | Mutagenesis |
| QC3_ACE2_H34UAG | gtcttcggcttcCtAgttaaacttgtc | Mutagenesis |
| QC5_ACE2_Y41UAG | gaagacctgttctaGcaaagttcacttg | Mutagenesis |
| QC3_ACE2_Y41UAG | caagtgaactttgCtagaacaggtcttc | Mutagenesis |
| QC5_ACE2_Q42UAG | gacctgttctatTAGagttcacttgc | Mutagenesis |
| QC3_ACE2_Q42UAG | gcaagtgaactCTAatagaacaggtc | Mutagenesis |
| QC5_ACE2_L79UAG | cagtccacaTAGgcccaaatg | Mutagenesis |
| QC3_ACE2_L79UAG | catttgggcCTAtgtggactg | Mutagenesis |
| QC5_RBD_K417N | acagacaggcaaCatcgccgactac | Mutagenesis |
| QC3_RBD_K417N | gtagtcggcgatGttgcctgtctgt | Mutagenesis |
| QC5_RBD_K458N | ctgttccggaaCtccaatctgaagc | Mutagenesis |
| QC3_RBD_K458N | gcttcagattggaGttccggaacag | Mutagenesis |
| QC5_RBD_K458H | gctgttccggCACtccaatctgaag | Mutagenesis |
| QC3_RBD_K458H | cttcagattggaGTGccggaacagc | Mutagenesis |
| QC5_RBD_K458R | gctgttccggaGgtccaatctgaag | Mutagenesis |
| QC3_RBD_K458R | cttcagattggacCtccggaacagc | Mutagenesis |
| QC5_RBD_Y473F | ccaccgagatctTtcaggccgg | Mutagenesis |
| QC3_RBD_Y473F | ccggcctgaAagatctcggtgg | Mutagenesis |
| QC5_RBD_Y489F | ggcttcaactgctTcttcccactgc | Mutagenesis |
| QC3_RBD_Y489F | gcagtgggaagAagcagttgaagcc | Mutagenesis |
| QC5_RBD_K417N | acagacaggcaaCatcgccgactac | Mutagenesis |
| QC3_RBD_K417N | gtagtcggcgatGttgcctgtctgt | Mutagenesis |
| QC5_RBD_K458N | ctgttccggaaCtccaatctgaagc | Mutagenesis |
| QC3_RBD_K458N | gcttcagattggaGttccggaacag | Mutagenesis |
| Synthetic fragment encoding C-terminal region of FSYRS | ctcgagcgtgaaattaccaggttctttgtggacaggggttttctggaaataaaatccccgatcctgatccctcttgagtatatcgaaaggatgggcattgataatgataccgaactttcaaaacagatcttcagggttgacaagaacttctgcctgagacccatgcttATTccaaacctttacaactacctgcgcaagcttgacagggccctgcctgatccaataaaaatttttgaaataggcccatgctacagaaaagagtccgacggcaaagaacacctcgaagagtttaccatgctgACCttcATCcagatgggatcgggatgcacacgggaaaatcttgaaagcataattacagacttcctgaaccacctgggaattgatttcaagatcgtaggcgattcctgcatggtcTTAggggatacccttgatgtaatgcacggagacctggaactttcctctgcagtagtcggacccataccgcttgaccgggaatggggtattgataaacccAAGataggggcaggtttcgggctcgaacgccttctcaaggttaaacacgactttaaaaatatcaagagagctgcaaggtccGAAtcttactataacgggatttctaccaacctgtaa | Subcloning |

*Upper-case letters indicate base substitutions introduced in the original sequences.
